# Origins of the Apple: The Role of Megafaunal Mutualism in the Domestication of *Malus* and Rosaceous Trees

**DOI:** 10.3389/fpls.2019.00617

**Published:** 2019-05-27

**Authors:** Robert Nicholas Spengler

**Affiliations:** Paleoethnobotany Laboratories, Department of Archaeology, Max Planck Institute for the Science of Human History, Jena, Germany

**Keywords:** domestication, apples, Rosaceae, mutualism, megafauna, seed dispersal, arboriculture

## Abstract

The apple (*Malus domestica* [Suckow] Borkh.) is one of the most economically and culturally significant fruits in the world today, and it is grown in all temperate zones. With over a thousand landraces recognized, the modern apple provides a unique case study for understanding plant evolution under human cultivation. Recent genomic and archaeobotanical studies have illuminated parts of the process of domestication in the Rosaceae family. Interestingly, these data seem to suggest that rosaceous arboreal crops did not follow the same pathway toward domestication as other domesticated, especially annual, plants. Unlike in cereal crops, tree domestication appears to have been rapid and driven by hybridization. Apple domestication also calls into question the concept of centers of domestication and human intentionality. Studies of arboreal domestication also illustrate the importance of fully understanding the seed dispersal processes in the wild progenitors when studying crop origins. Large fruits in Rosaceae evolved as a seed-dispersal adaptation recruiting megafaunal mammals of the late Miocene. Genetic studies illustrate that the increase in fruit size and changes in morphology during evolution in the wild resulted from hybridization events and were selected for by large seed dispersers. Humans over the past three millennia have fixed larger-fruiting hybrids through grafting and cloning. Ultimately, the process of evolution under human cultivation parallels the natural evolution of larger fruits in the clade as an adaptive strategy, which resulted in mutualism with large mammalian seed dispersers (disperser recruitment).

## Introduction

The early cultivation of long-generation arboreal crops is one of the least explored frontiers in plant domestication studies. The mystery of tree domestication is further compounded by genetic studies (e.g., Gross et al., 2014a; Velasco et al., 2016), which have demonstrated that stochastic evolutionary forces, notably bottlenecks and subsequent diversification, were not major contributing factors to apple (*Malus domestica* [Suckow] Borkh.; *Malus pumila* Mill.) or peach (*Prunus persica* [L.] Batsch) evolution under cultivation. Additionally, the concept of “domestication” falls apart when discussing large-fruiting trees, which are hybrids expressing exceptional growth or heterosis, and the introgression of traits is often not fixed. Planting an apple seed will result in a tree that may express any of a variety of characteristics, and in many cases, fruit morphology of feral trees resembles progenitors. Hybrid fruit trees do not represent “domestication” the same as domesticated grasses; the introgression of traits is often not fixed and only maintained through cloning. Likewise, traits associated with fruit shape, size, sugar content, color, and texture are often highly plastic or variable; the role of developmental plasticity in these trees is poorly understood. Wild Tian Shan apple trees can produce fruits up to 8 cm in diameter, overlapping in size with some modern landraces. Ultimately, the rule book for domestication needs to be rewritten when dealing with perennials, especially long-generation arboreal crops. As Gross et al. (2014a,b); also Fuller (2017) noted, the origins of our fruit trees remain one of the biggest unanswered questions in domestication studies. In this article, I compile recent genetic, paleontological, and archeological data in order to present a model to explain the rapid evolution of rosaceous fruits under human cultivation. This model for evolution under cultivation focuses on the greater time depth of evolution and a robust understanding of gene-flow/seed-dispersal processes before and during human cultivation. In this article, I review domestication in the *Malus* genus, but relate it to what appear to be parallel processes across the Rosaceae family, in order to critique growing trends in domestication studies.

Plant domestication studies over the past 15 years have seen a theoretical shift, including the rejection of the idea of a rapid Neolithic Revolution in favor of protracted models for domestication (Tanno and Willcox, 2006; Fuller et al., 2010, 2011, 2012). The new protracted models suggest that domestication took two to three thousand plant generations for the first trait – tough rachises – to fully introgress into cultivated populations (Fuller et al., 2012). Likewise, scholars continue to debate whether we can discuss centers of domestication or if the process occurred over a large geographic area, possibly in parallel across multiple areas (Langlie et al., 2014). Other ongoing debates in the field include questions over whether humans were consciously directing evolution and what constitutes a fully domesticated plant, especially when discussing shifting allele frequencies in a larger population. One of the major shortcomings of these revisions of domestication theory is the prominent focus on three large-seeded grasses – wheat (*Triticum* spp. L.), barley (*Hordeum vulgare* L.), and rice (*Oryza sativa* L.). The revisions largely ignore the thousands of other domesticated plants growing around the world today, many of which followed different pathways toward domestication. In some cases, the domestication process was rather rapid (e.g., polyploidy or hybridization). The apple has a generation span of roughly 20 years and has been cultivated by humans for only three to four millennia; hence, there simply has not been the two to three thousand generations that a protracted model would require. Furthermore, most apple propagation over the past two millennia has relied on cloning and grafting, significantly reducing further the already narrow temporal window for evolution to occur. Current archaeobotanical evidence seems to suggest that apple domestication took place over a period of less than 100 generations, much less for the earliest morphological changes. It seems feasible that rapid domestication through hybridization occurred in as little as one or a few generations, and most of the modern diversity in landraces is probably a recent phenomenon, through directed breeding. Not only do protracted models of domestication fall short when discussing apples, the concept of a “center” of domestication is misleading. Genetic studies illustrate that wild apple populations across Europe and West Asia collectively contributed to the modern domesticated apple in a hybrid complex of species distributed across a continent and a half (Cornille et al., 2014, 2015).

### Gene Flow Through Seed Dispersal

Gene flow, both intra- and inter-species, is necessary for ensuring a healthy population, promoting adaptation, diversification, and evolution (Ellstrand et al., 1999; Garant et al., 2007; Feder et al., 2012; Ellstrand, 2014). Density-dependent mortality forces plants to evolve ways to disperse their seeds, especially for large-seeded, large-fruiting trees (Chapman and Chapman, 1995; Wills et al., 1997; Harms et al., 2000). Large apple fruits often do not fall far from the tree and will be over shadowed and out competed by the parent unless the fruit is consumed and the seeds are transported. Competition and predation on offspring would have provided strong selective pressure to evolve successful dispersal strategies (Janzen, 1970; Connell, 1971; Kellner and Hubbell, 2018). Seed-dispersal provides plants with mobility in an evolutionary sense, helping them avoid kin competition, allowing for the colonization of new areas, and facilitating escape from high population density or poor growing conditions (Hamilton and May, 1977; Holt et al., 2005; Ashley, 2010). Likewise, both seed and pollen dispersal help reduce inbreeding depression and maintenance of a strong genetic population (Greenwood et al., 1978; Waser et al., 1986; Nathan and Muller-Landau, 2000; Jara-Guerrero et al., 2018).

Biotic dispersal can also lead to directed dispersal, targeting prime colonization areas (Eriksson, 2008). For example, apple trees need to continually colonize new open patches as over-canopy trees grow to dominate areas near parent trees; therefore, recruiting animal dispersers that live or feed in these open patches ensures that seeds are dispersed directly to optimal areas for colonization. Evolutionary studies of large fruit development, based on the fossil record, show that increases in seed size or energy allotted to fruit production directly correlate with a reduction in the number of seeds produced or energy available for other plant functions (Moles et al., 2004; Eriksson, 2008). Therefore, the evolution of larger energetically costly fruits was likely accompanied by strong selective pressure for seed dispersal. The heavy metabolic investment in sugar production likely correlates with the recruitment of an animal disperser.

The apple fruit is an impressive evolutionary adaptation for seed dispersal. As with most domesticated plants, the key to understanding domestication in plants with large fruits rests in a switch from the wild to an anthropogenic seed-dispersal mechanism. Many domesticated crops either possessed weak dispersal strategies in the wild, as is the case in large-seeded cereals (Wood and Lenné, 2018), or had lost their natural seed-dispersal mechanism before humans started more heavily interacting with them, as is the case for many large fruits (Janzen and Martin, 1982; Kistler et al., 2015) or small-seeded grains (Spengler and Mueller, in press). The lack of, or weak, gene-flow regimes in these plants before human intervention left them with a predisposition toward domestication, especially if fragmentary and genetically isolated populations were crossed/hybridized for the first time since the last glacial advance. Cornille et al. (2014, 2015) have published several detailed studies that illustrate a relatively genetically heterogeneous population in wild apples across Eurasia, and many studies have noted particularly weak gene flow regimes in the Tian Shan wild apples. Ultimately, understanding the evolution of seed-dispersal-based mutualism in the wild among trees with large fruits is the key to understanding how these fruits evolved in response to human seed-dispersal processes.

## Large Fruits and Megafaunal Seed Dispersal

### Recruitment of Megafaunal Dispersers

Fleshy fruits are usually adaptations for animal dispersal (endozoochory); large fruits are usually adaptations for large mammalian dispersal. I refer to megafauna as any animal, extant of extinct, larger than 40 kg. Many paleontologists now recognize that the seed dispersers for plants that produce large fleshy fruits, such as cucurbits and many trees, were now-extinct megafaunal herbivores (Janzen and Martin, 1982; Kistler et al., 2015). Barlow (2002); following Janzen and Martin (1982) referred to these large fruits as evolutionary anachronisms. Notably, paleontologists working in the tropical forests of South America have argued that these large-seed dispersers were gomphotheres and other Pleistocene megafauna (Janzen and Martin, 1982). Large fruits in most ecosystems around the world were likely more effectively dispersed before the extensive megafaunal extinctions of the Late Pleistocene. While many megafaunal mammals still exist today and some maintained relatively high densities into the early Holocene, the densities of large mammals on at least five continents were much higher before the onset of the Holocene (Rule et al., 2012; Faurby and Svenning, 2015; Pires et al., 2017). Large-fruiting wild apples (*Malus dasyphylla* Borkh. *M. niedzwetzkyana* Dieck ex Koehne, *M. orientalis* Uglitzk., *M. sieversii* [Ledeb.] M. Roem., and *M. sylvestris* Mill.) and wild peach and plum relatives (*Prunus armeniaca* L., *P. persica* [L.] Batsch, *P. mira* Koehne, and *P. davidiana* [Carrière] Franch.) also have physiological traits that seem to suggest coevolution with megafaunal mammals. Coevolutionary processes are not always easy to understand based on modern biotic communities, and mutualistic relationships, especially relating to seed dispersal, often involve guilds of disperser species, many of which are extinct (Jaroszewicz, 2013; Escribano-Avila et al., 2014). Seed dispersal mutualism usually relies on guilds of animals, what Tiffney (2004) refers to as diffuse coevolution (Janzen and Martin, 1982; Tiffney and Mazer, 1995; Wenny, 2001). Large-fruiting Rosaceae trees, especially in the *Malus* clade, have found continual seed dispersal from opportunistic mammalian dispersers, such as humans, bear, and possibly deer.

Trees with large fruit and smaller seeds, such as apples, can escape rapid population loss after the extinction of their primary dispersers, due to some continual seed dispersal by smaller omnivorous mammals or opportunistic browsers (Guimarães et al., 2008). The small seeds in *Malus* fruits allow them to more effectively disperse by means of extant mammals, including mid-sized omnivores and bears. The key to understanding population distribution of wild apples in the Holocene is a reduction in the densities of dispersers, rather than a complete loss of dispersers. Jara-Guerrero et al. (2018) demonstrate that some deer species continue to disperse certain megafaunal-dispersed trees, in their study these trees mostly produced non-sugary legume pods. Better post-digestion germination studies are required for *Malus* spp. and deer. Deer have more restrictive digestive systems than many other artiodactyla, and the survival rates of seeds post-digestions is unknown.

A recent study by Onstein et al. (2018) illustrated both a decrease in size and an increased rate of extinction of megafruits in the Arecaceae family during the Holocene in response to the loss of megafaunal dispersers. While palms are not direct analogs for dicotyledonous trees, the trends between species with large fruits appear to be similar. Other studies have demonstrated that the loss of megafaunal dispersers resulted in a loss of seed dispersal and subsequently extinction or fragmentation of large-fruiting plant populations (Galetti et al., 2006, 2017; Eriksson, 2008; Malhi et al., 2016; Pires et al., 2017). The extinction of their seed dispersers and consequently low gene flow caused major changes in the population genetic structure among most large-fruiting trees (Guimarães et al., 2008). In tropical South American large-fruiting tree populations, a severe bottleneck and a reduction in genetic variation is coincident with megafaunal extinction. Many trees evolve so closely with their disperser that seed coats prevent germination without acid scarification through digestion (Kleyheeg et al., 2018). Experiments with horses, clearly demonstrate the viability of certain seeds after digestion – Janzen and Martin’s (1982) original study demonstrated a 97 percent post-digestion germination rate. Field studies have shown that in some fruit species dormancy is never broken without scarification through digestion (Howe and Smallwood, 1982). Spengler and Mueller (in press) recently illustrated how breaking of endozoochoric seed-dispersal mutualism through the evolution of new seed traits is the key to domestication in many plant species.

Data show that most megafaunally dispersed fruits experienced range contractions starting in the early Holocene (van Zonneveld et al., 2017). These studies also show that there were reductions in genetic variability among these populations and strong implications for the broader ecosystem, including carbon storage and nitrogen transport (Doughty et al., 2016; Malhi et al., 2016). Megafruits in both temperate and tropical ecosystems have continued to lose range throughout the Holocene and many are endangered. Primates, certain birds, and large frugivorous mammals have retained some of the megafruit diversity in certain tropical forests, but these dispersers are absent or rare in temperate zones today. The idea of a “partial loss of dispersal agents” (Janzen and Martin, 1982, p. 25) is important when understanding apple populations in West Asia and Europe today. Many of the megafaunal-dispersed trees that still exist today have slowed the process toward extinction by implementing vegetative sprouting and asexual reproduction, relying on human seed dispersal, and the occasionally dispersed seed via gravity or flood waters. van Zonneveld et al. (2017) demonstrated that humans have maintained some of the current ranges for many megafaunal-dispersed fruit trees, although often greatly reduced.

### The Evolution of Megafruits

The fossil record demonstrates that many of these large fruits evolved millions of years ago, and therefore, the faunal record of that period contains the mutualistic companion(s). Most examples of megafauna-dispersed fruits share a similar suite of traits – a megafaunal dispersal syndrome (first described by Janzen and Martin, 1982). Several examples of megafruits have persisted in temperate zones of the Northern Hemisphere, despite dramatic losses of their dispersal fauna during the terminal phase of the Pleistocene. A few examples of megafruits from North America include Osage orange (*Maclura pomifera* [Raf.] C.K. Schneid.), persimmons (*Diospyros* spp.), pawpaws (*Asimina triloba* [L.] Dunal), and several large Fabaceae (e.g., *Gleditsia triacanthos, Gymnocladus dioicus*); from Western Europe megafruits include several species of *Prunus* with stones too large for avian or small-mammalian dispersal and wild large-fruiting *Malus* spp., *Mespilus germanica* L., and *Pyrus pyraster* (L.) Burgsd. Megafruits of temperate Asia also include a number of *Prunus* species, notably the apricot (*Prunus armenica*) and peach (*P. persica*), as well as persimmons, *Cydonia* sp., *Eriobotrya* sp., and jujube (*Ziziphus* spp.).

**FIGURE 1:**
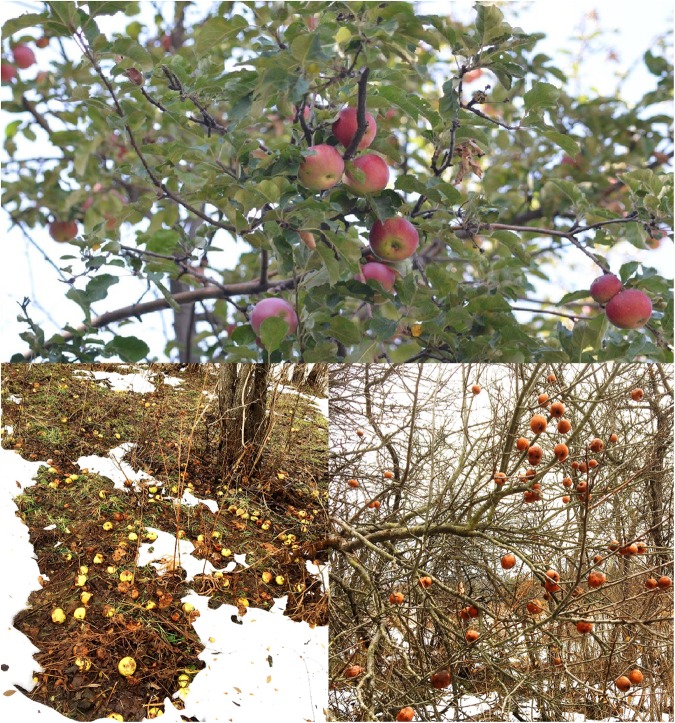
Top: wild Tian Shan apples retained by the tree after the fruit reaches ripeness; photo taken by Martin R. Stuchtey; Bottom: Feral apples rotting under trees in a cow pasture in New York State. Near complete generation losses are often observed when rotting fruit cause seed death through alcohol buildup and fungal attack and rodents pray on seeds, attracted by concentrations of food sources. Likewise, any offspring that do germinate directly below the parent tree will be out competed. The extreme energy investment in seed dispersal would not be repaid in temperate Northern Hemisphere regions without human intervention. Many of these feral trees express traits of wild apples, such as retaining their fruits after ripeness, seen in the figure to the right variable sugar production and smaller fruits.

Dropping fruit directly below a tree leads to parent/offspring competition, sibling/sibling competition, attraction of seed predators due to heavy densities of food, and results in the rotting of fruits with high sugar concentration. Following the Janzen-Connell Hypothesis, density-dependent mortality would have provided a strong selective force for plants to evolve traits that attracted animals to disperse their seeds (Janzen, 1970; Connell, 1971; Kellner and Hubbell, 2018). Fruit rotting, in many cases, causes seed destruction through fungal attack and fermentation of the sugars. Rodents (including squirrels) will chew through the fruit to eat the seeds. Many wild and feral apple populations retain their fruit well after the seeds are ripe and the sugar content is at its highest – this retention of fruit suggests that the trees evolved with a disperser able to reach terminal limbs. Ungulates rarely feed on apples, likely due in part to the acidity and in part to the double digestion – highly sugary foods cause irritation as they ferment/are consumed by microbiota in the stomach (as evidenced by high methane levels from corn-fed cows). Schnitzler et al. (2014) noted that apples are often found in abundance rotting under apple trees in cow pastures (Figure 1). Buttenshøn and Buttenshøn (1999) noted that apple seeds are more likely to be destroyed if the fruit is consumed by a ruminant grazer, as opposed to a monogastric browser. Likewise, endozoochoric studies of ruminant digesters illustrate that they do not readily pass viable seeds over 2.0 mm in diameter (Wallace and Charles, 2013). The restrictive digestive system of ruminants retains larger plant material for double digestion, destroying large seeds, and, presumably, the thin seed coat of an apple would make it particularly susceptible. Perissodactyla (including horses, rhinoceroses, and tapirs) are far more likely to disperse large seeds and consume sugary fruits than true ruminants. Artiodactyla (including cattle, deer, and their relatives) are more readily featured in zoochory studies (Nathan et al., 2008; O’Farrill et al., 2013; cf. Jara-Guerrero et al., 2018), likely due to the high terminal Pleistocene extinctions of temperate Northern Hemisphere Perissodactyla. In Africa where the mass megafaunal extinctions of the late Pleistocene did not occur, dispersers include elephants, equids, certain gazelle, primates, and rhinoceroses (Dinerstein, 1991; Feer, 1995).

Paleontologists have noted that the establishment of an intercontinental land bridge between Europe and Africa around 19 million years ago led to the dispersal of several African faunal clades northward. These clades included proboscideans (*Deinotherium*), perissodactyla (*Chalicotherium*), artiodactyls (suids and *Dorcatherium*), aardvarks (*Orycteropus*), and catarrhine primates (*Griphopithecus* and *Pliopithecus*; Begun et al., 2012). Interestingly, many of these clades are fructivores and their gradual spread into Eurasia appears to have been accompanied by the dispersal of African forests and fruit trees, as well as the evolution of new plants with large fruits. The early Miocene land bridge had a bi-directional flow, and ungulates, such as giraffes and bovids, spread from the Eurasian grasslands into Africa (Steininger et al., 1985; van der Made, 1999). The Eurasian large-mammal clades of the early Miocene were largely grazers with flat grinding teeth; fruiting Rosaceae were mostly short shrubs with small fruits well-adapted to avian dispersal. Once these African clades of fruit eaters spread into Eurasia, the vegetation communities changed and many plant species began to evolve in response, most notably by developing larger endozoochoric dispersal propagules. The fossil record relating to early and mid-Miocene Europe suggests that fleshy-fruit-based endozoochory was rare on the primarily savanna landscape (Fortelius et al., 2006). A drop in overall seed size in the European fossil flora through the Miocene likely correlates with a loss of forests and opening of the grasslands (Eriksson, 2008).

## Fruit Evolution in Rosaceae

### Understanding Extant Populations

**FIGURE 2:**
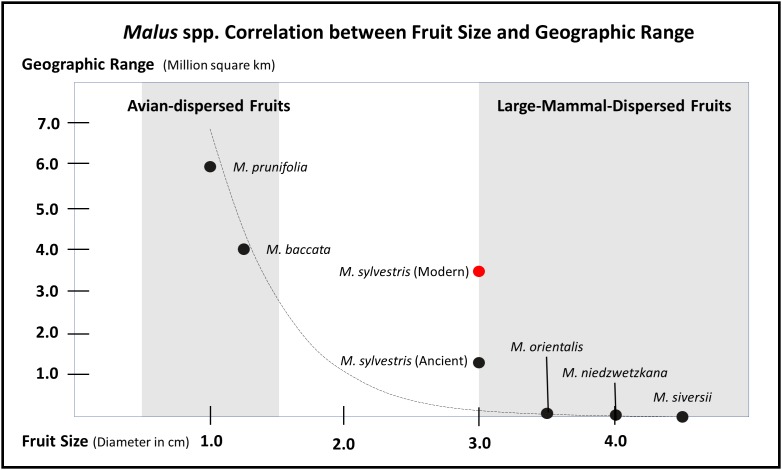
Graph illustrating how fruit size inversely correlates with modern distribution ranges in the genus *Malus*. This correlation is likely a result of large-fruiting trees losing geographic range throughout the Holocene, after the density of megafaunal dispersers decreased. The exponential trend curve is calculated based on the estimated early Holocene range for *M. sylvestris* and not the modern range (see Figure 3, 4). This table is meant to show a general trend and ecologically constrained species complicate the trend, notable endemic crabapples do exist with smaller ranges. Likewise, there is often lack of consensus over range and taxonomy of species in this genus; some scholars claim *M. baccata* is restricted to Siberia and other place it south all the way to Nepal and Bhutan. This general trend of large-mammal-dispersed fruits having small geographic ranges, genetically heterogeneous populations, and low rates of colonization also holds up for *Malus, Pyrus, Cydonia, Mespilus*, and *Prunus*; whereas, clades such as *Rubus* and *Crataegus* contain species with large geographic ranges. This table is highly conservative, if other small-fruiting Rosaceae with larger ranges were added, the tail on the *Y*-axis could extend considerably; likewise, acknowledging that the Tian Shan wild apples can have diameters up to 8 cm would considerably extend the tail on the *X*-axis. The distribution ranges and fruit diameters presented here are estimated based on descriptions in the *Flora Europeae* and the *Flora of China*.

Rosaceae contains a diversity of fruit forms, which have evolved for an equally diverse array of dispersal strategies. The *Flora Europaea* recognizes 35 genera in the family, many of which are herbaceous; of the woody clades, the vast majority are shrubby with small avian-dispersed fruits (Webb et al., 1964). Fleshy fruits are an evolutionary adaptation for endozoochory (dispersal through animal ingestion), and they evolved at least twice in two distinct subfamilies. Large-fruiting species evolved independently in both the apple and peach subfamilies (Wu et al., 2013; Xiang et al., 2016). Like large-fruiting species in the American tropics (Doughty et al., 2016; Jara-Guerrero et al., 2018; Onstein et al., 2018), Eurasian large-fruiting rosaceous species show clear signs of range reduction and loss in genetic diversity throughout the Holocene. Many, wild large-fruiting rosaceous trees exist in fragmentary populations across Europe and Asia and, in most cases, are listed as endangered (e.g., *Prunus americana*). It is informative that the larger the fruit among trees in the Rosaceae family, the more restricted the extant range. In Figure 2, I illustrate that there is a correlation between fruit size and distribution range in European species of *Malus*; a similar rough correlation exists for Asian species of *Prunus*. It is also informative to note that out of all extant trees/shrubs in Rosaceae with fleshy fruits, the vast majority are avian dispersed. Similarly, avian-dispersed clades express far greater diversity and range coverage than mammalian-dispersed clades. For example, the *Flora Europaea* recognizes 74 species of *Rubus*; although, many members of the genus readily hybridize creating a hybrid complex that covers much of Eurasia. The flora recognizes 23 species of *Crataegus*, 11 in *Cotoneaster*, and 18 in *Sorbus*. Contrasting that to the few extant large-mammal-dispersed clades recognized by the flora, *Mespilus* contains a single species restricted to a small range of Central Europe before humans spread them as ornamentals. *Cydonia* also only has a single species and was likely restricted in range before human intervention. While the flora recognized 6 *Malus* species, at least two have been taxonomically collapsed and only two or three of the remaining species produce large fruits – again with restricted ranges before human intervention.

Wild apple trees are a low-growing species, and they express low competitive ability when confronted with larger canopy species. They are naturally scattered in patches in openings and at the edges of forests and areas of early forest succession (Schnitzler et al., 2014). Wild apple populations today are largely reliant on rapid colonization of forest openings or patchy landscapes, and *Malus* spp. would have been well-suited to the largely open savannas of the European late Miocene. This habit leads to strong selective pressure for seed dispersal to colonize newly opened forest patches and for dispersal agents that are not forest dwellers. Due to the continually dynamic nature of these small-scale forest clearings, long-distance seed dispersal, especially directed to suitable colonization areas would appear to benefit wild apples. Many woody Rosaceae have evolved this habit of pioneering forest openings; *Amelanchier, Cotoneaster, Crataegus, Pyrus, Pyrachanthus, Rosa, Rubus, Sorbus*, and many *Prunus* and *Malus* species all express an avian-dispersal syndrome. Therefore, the loss of the avian-dispersal mechanism through the evolution of larger fruits was likely driven by selection for a more effective disperser. The larger fruiting clades likely evolved with dispersers that foraged at forest edges and had large foraging ranges. The larger the fruit, the more strictly endozoochoric the plant becomes, and trees with large fleshy fruit rely on their dispersers for the reproductive success of the species.

### The Early Fossil Record

Plants in Rosaceae were prominent across North America and present in Eurasia during the Eocene (56.0 to 33.9 M years ago), and an extensive fossil record exists (see DeVore and Pigg, 2007). European fossil beds, such as the Messel Pit in Germany, illustrate the presence of members of the family; although, in extremely low abundance and with limited diversity, restricted to low-growing plants with small fruits (Collinson et al., 2012). Fossils from across Asia and Europe of early and mid-Eocene *Prunus* endocarps are small (Dorofeev, 1963; Kirchheimer, 1973; Mai, 1984, 1995; Li et al., 2011). DeVore and Pigg (2007) claim that the fossils of Eocene and Miocene *Prunus* pits from North America, Asia, and Europe are all comparable in morphology to modern wild cherries. Other small-fruiting Eocene rosaceous fossils include *Rubus, Crataegus*, and *Sorbus*; *Sorbaria* was restricted to Asia; and *Pyracantha* in Europe and Asia (DeVore and Pigg, 2007). Members of this clade increase in prominence and diversity in the fossil record of the Miocene. The Miocene fossil record for Rosaceae in Europe has been synthesized by Kirchheimer (1973); also see Mai and Walther (1978); Mai (1984, 1995); Kvaček and Walther (1998, 2001, 2004); DeVore and Pigg (2007). There are some reports of Eocene leaves that resemble *Malus/Pyrus*, including leaves from North America, at the fossil site nicknamed the “Eocene Orchards” (Wehr and Hopkins, 1994). There is a much clearer fossil record for Rosaceae fruits and seeds from Europe and Asia during the Miocene (20.0 to 5.3 M years ago) and Pliocene (5.3 to 2.6 M years ago; Dorofeev, 1963; Mai and Walther, 1978; Kvaček and Walther, 1998, 2001, 2004; Martinetto, 2001). By the Miocene, the radiation of the family, led to specimens that morphologically resemble *Rosa, Crataegus, Mespilus, Potentilla, Cotoneaster*, and *Agrimonia* across Europe, and *Prunus, Sorbus*, and *Rubus* species remained common. Clades, such as *Crataegus, Prunus, Amelanchier*, and *Pyrus* formed in North America at this time. Many of these clades and several distinct morphological features appear to have risen out of hybridization events. In Asia, *Prunus, Rosa*, and *Sorbus* are common fossils from Miocene and Pliocene strata, and many of these genera spread into Africa around the same time that African megafauna spread into Europe (see DeVore and Pigg, 2007).

Following the mid-Miocene Climatic Optimum (ca. 14 M years ago), Rosaceae diversified likely in response to increasingly more arid climatic conditions (Töpel et al., 2012). As larger fruits evolved in the independent subfamilies of Amygdaleae and Maleae, tree-form growth habits appear to have also evolved, producing more photosynthetic leaves to support the production of larger fruits. In North America, decreasing sea levels as a result of glaciation of Antarctica eventually led to dryer summer conditions by the mid-Miocene, and a Mediterranean-type climate with dry summers and winter precipitation came to characterize much of North America and Europe (Töpel et al., 2012). Numerous lines of paleoclimatic data all collate to depict a cool semi-arid grassland ecology covering much of Eurasia during the late Miocene (Jacobs et al., 1999; Ivanov et al., 2002, 2011; Agustí et al., 2003; Bruch et al., 2006; Fortelius et al., 2006; Pickford et al., 2006; Agustí, 2007; Akgün et al., 2007; Strömberg et al., 2007; Kovar-Eder et al., 2008). Fossil records are highly fragmentary for fruit evolution, but when combined with genetic data, it seems likely that large-fruiting trees in the Rosaceae family date at least as far back as the Pliocene, more likely the late Miocene (11.6 to 5.3 M years ago). They, therefore, evolved to recruit an animal on a very different landscape than that of modern Eurasia. Genetic studies show that the diversification of the pome-fruiting rosaceous clades, notably *Pyrus* and *Malus*, took place in the late Miocene (Töpel et al., 2012). While the larger fruit sizes in certain species within these clades may have evolved after diversification, it seems likely that the large fruits were a response to faunal dispersers of the late Miocene through the Pliocene of Eurasia. Interestingly, the large-fruiting clades of *Prunus*, notably *P. persica*, appear to have diversified and separated from the broader Amygdaleae lineage around the same time (Velasco et al., 2016). If we assume that almonds and peaches shared an ancestor with large fruits, then genetic studies would suggest that large fruits in the Amygdaleae clade evolved over seven million years ago (Velasco et al., 2016).

### Hybridization and Evolution

Fruit morphology in the Rosaceae family arose through a series of polyploidy events, including a whole genome duplication that appears to have given rise to the *Malus* clade and its large pomes (Xiang et al., 2016). A whole genome duplication may also be responsible for the development of large-fruiting members of the *Prunus* group. Wu et al. (2013) suggested that a whole genome duplication was shared by all *Pyrus* and *Malus* species. The leading theory for the formation of the Maleae subfamily is a hybrid event with an ancestral member of the Spiraeoideae and Amygdaleae subfamilies (described in Xiang et al., 2016). The 17-base chromosome counts of all members in the subfamily is usually used as the main line of reasoning for an argument of a whole genome duplication at the base of the clade (Dickinson et al., 2007). However, subsequent hybridization events likely resulted in the diversity of fruit forms seen in the family today, which range from dry to fleshy, with inferior to superior ovaries, and small to large-sized fruits. In addition, members of the family, such as *Fragaria* and *Rubus* produce aggregates of fruits, in the case of *Fragaria*, dry fruits. Whereas, the drupes in *Prunus* have one fertilized ovule and most Roseae and Maleae species have five.

Phylogenetic studies based on nuclear DNA have shown that the drupes in *Prunus* evolved twice from a follicetum, that pomes evolved from a coccetum, which originally evolved from folliceta, and that multiple drupelet clusters evolved twice (Evans and Campbell, 2002; Xiang et al., 2016). While Rosaceae was a recognizable clade, as far back as the early Eocene, it really did not diversify or become abundant for the first fifty million years of its existence. The fossil and genetic evidence suggests that the pome-producing clade started to diversify in the early Oligocene, but these were small-fruit producing plants (Xiang et al., 2016). The large-fruiting forms of *Malus, Pyrus*, and *Cydonia* appear to have evolved in the late Miocene and through the Pliocene. Ultimately, the thick flesh of *Malus* fruits, their one or two-seeded carpels, and the centrally located endocarp are all derived features of the lineage. The fully inferior ovary is a derived trait in fruits of the *Sorbus, Pyrus*, and *Malus* clades. Hybridization in this clade is supported by a lack of interspecific genetic barriers and self-incompatibility. Likewise, isolated populations result from low rates of gene flow, especially from seed dispersal, leading to extended periods of genetic isolation. The crossing of specimen from these isolated populations appears to result in heterosis or unique offspring. High levels of phenotypic plasticity also lead to considerable variability in features, such as fruit size, which can be greater expressed in hybrids and maintained by humans through cloning. Understanding the evolutionary processes that drove morphological changes of fruit form and size in the family in the wild is important for understanding how fruit size further increased under early human cultivation. The key for understanding phenotypical change in the Rosaceae clade is hybridization and fruit form is almost always directly linked to seed-dispersal mutualism.

### Climate Change and Glacial Refugia

Each glacial advance or aridification of Central Europe brought with it increasing pressure for plants to evolve more effective seed-dispersal mechanisms, especially ones that could move seeds across large expanses of grassland between environmental refugia (Clark, 1998). Maintaining gene flow and population integrity on a mosaic landscape ultimately drove selection for larger fruits to recruit larger seed dispersers. Climate changes from the late Miocene through the Pleistocene caused population fragmentation and range fluctuations among many plant species, with greater impacts on long-generation arboreal species. Vegetation communities across the Northern Hemisphere continually fragmented and migrated; this process resulted in hundreds of thousands to millions of years of genetic isolation between populations before being reconnected by melting ice or changes in forest cover (Clark, 1998; Taylor et al., 2015). Studies repeatedly illustrate the importance of hybridization in speciation (e.g., Hewitt, 1988; Harrison, 1993; Abbott et al., 2013; Taylor et al., 2015). Hybridization zones today tend to cluster in recolonization areas between glacial refugial pockets or, in some cases, in disturbed anthropogenic landscapes (Jiggins et al., 1996; Swenson and Howard, 2005). Taylor et al. (2015) demonstrate that changes in hybrid zones are usually representative of climate change, this is relevant today with increasing anthropogenic climatic shift and it was the case at the Pleistocene/Holocene boundary. Population genetic studies for several of our more familiar arboreal crops suggest that they experienced limited gene flow through the Holocene and may have existed as discrete populations through much of the Pleistocene (e.g., Pollegioni et al., 2017). These genetically isolated populations illustrate that trees could not cross glacial barriers during peak glaciation, but also that seed dispersal over the past 13,000 years has been limited in range.

Megafruits of the northern temperate zones today usually have highly fragmented populations and are often endangered. Without the full guild of their natural seed dispersers, their rates of gene flow have dramatically decreased – in some cases, such as in the Tian Shan apples or the Osage orange, to the point where they were restricted to a few fragmented populations of densely clustered individuals that cannot disperse their offspring. The trees of *M. sieversii* are 2 to 10 m tall with yellowish green fruit that develop a reddish hue when ripe (Omasheva et al., 2017). They are often located on steep slopes between 800 and 1500 masl, likely restricted in range to areas where heavy herd-animal grazing does not stunt new growth (Dzhangaliev, 2003). However, their isolation on hill slopes suggests that they cannot maintain a population relying only on gravity-mediated seed dispersal. Omasheva et al. (2017) noted that there is a significant degree of gene flow from domesticated to wild apples in the wild range of the Tian Shan. They also noted that the population is threatened with extinction and is losing genetic integrity. Limited gene flow within the wild apple population illustrates limited seed dispersal, despite continual pollen dispersal.

The natural range of *M. sieversii* covers the western extent of the Tian Shan Mountains and is mostly restricted to southeastern Kazakhstan, with isolated populations in valleys in northern Kyrgyzstan, Tajikistan, and Uzbekistan (Harris et al., 2002; Velasco et al., 2010; Cornille et al., 2014; Figure 2). A genetically isolated ecotype has also been noted in far western Xinjiang (Duan et al., 2017). Many scholars studying the Central Asian wild apple populations have noted distinct ecologically defined populations or ecotypes; these may, in part, arise through the extreme plastic responses in the clade. However, it is likely that these disparate populations, many of which used to be considered separate species by taxonomists (see Omasheva et al., 2015), are genetically isolated groups within a restricted range of distribution. *Malus niedzwetzkyana* is still considered its own species by many botanists and has a slightly larger range of distribution than other large-fruiting *Malus* trees in Central Asia, stretching south all the way to the Afghanistan border (Omasheva et al., 2015). Population genetics studies of the core population in southeastern Kazakhstan (Figure 2) identified three distinct genetically isolated population clusters (Omasheva et al., 2017). Richards et al. (2009) noted that there was likely a larger ancestral population covering the range of all of these now-isolated populations. While pollen in this self-incompatible line can lead to gene flow up to 10.7 km (Reim et al., 2015), the near complete lack of seed dispersal still prevents the colonization of new areas and maintains genetic barriers.

**FIGURE 3:**
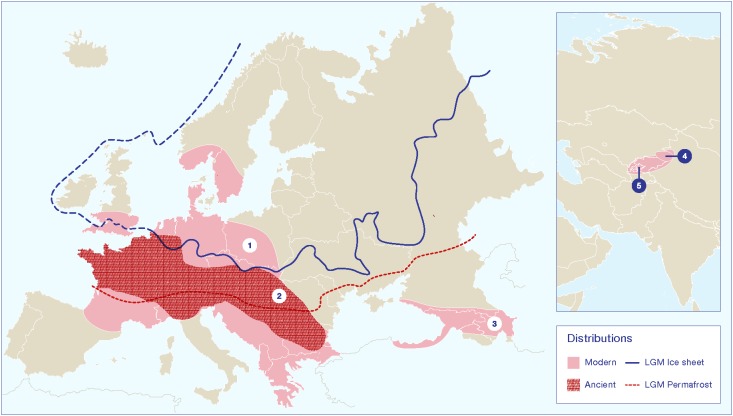
Map showing the modern distributions of key *Malus* species in relation to known Pleistocene glacial and permafrost limits during the Last Glacial Maximum (LGM). Distribution range (1) is an estimate of the modern range for *Malus sylvestris* based on description in the *Flora Europeae*. Note that the use of this species and related hybrids as ornamentals complicated estimates of a late Holocene range distribution. Distribution range (2) represents the estimated range of the European wild apple during the early and mid-Holocene, based on archaeobotanical data, as presented in Figure 4. Distribution range (3) represents an estimated range for *M. orientalis*, based on the *Flora Europeae*. Distribution range (4) is the core area of the Tian Shan wild apples, and range (5) is the broader distribution of closely related species/subspecies, including *M. niedzwetzkyana* and *M. kirghisorum*, which are now taxonomically clumped into *M. siversii* populations. Glacial and permafrost cover estimates for the LGM were made based on Davis (1976); Hewitt (1996); Hewitt (1999); Petit et al. (2003); Svendsen et al. (2004).

The European wild apple (*M. sylvestris*) appears to have hybridized with wild Tian Shan apples at some point over the past three millennia, creating what we think of as domesticated apples (Gross et al., 2012, 2013, 2014a,b; Cornille et al., 2014). Studies of the population genetics of the European wild apple have identified three distinct genetically isolated populations, which likely reflect Pleistocene refugia (Cornille et al., 2014, 2015). The scholars conducting these studies also discuss the effects of decreasing diversity in pollinator species; however, there have been few discussions of the seed dispersers. A constant rate of seed dispersal across the Pleistocene/Holocene boundary for wild *Malus* spp. is often assumed. Cornille et al. (2014) noted that European wild apples are endozoochoric; they also question why there is not a strong homogenous gene structure across the broader population. Ultimately, they conclude that the lack of a homogeneous genetic structure is a response to fragmentation during the Pleistocene and a narrow window of recuperation for the broader population during the Holocene. An impressive study of 1889 wild and 339 landrace apples from central Western Europe, depicted a genetically heterogeneous population with at least five distinguishable groups (Cornille et al., 2015). In this regard, the limited number of generations since the glacial retreat may not have been sufficient for populations to become homogeneous. However, they further note that long-distance dispersal is more likely in animal-dispersed trees, and that *Malus* species have shorter generations than most arboreal plant. The missing variable in the equation would appear to be a loss of the primary dispersers or a general reduction in the number of possible dispersers. It seems likely that the lack of diversification is a response to low gene flow combined with the limited time since the glacial retreat. The loss of the primary seed dispersers for the European wild apple led to reduced ability to colonize new areas during the early and mid-Holocene. As I illustrate in Figure 3, the distribution of archaeobotanical finds of apple seeds from Neolithic sites in Europe seems to suggest that the range for the wild apple was more restricted before the past three millennia.

Cornille et al. (2014) noted that there are hotspots of allelic richness for *M. sylvestris* dispersed across Europe; these hotspots, in theory, map to areas of Pleistocene ice-free refugia and spots with healthy seed-disperser populations. Wild-apple hotspots match known glacial refugia and include central Germany, southern France, central Italy, the Balkans, and the Carpathian Mountains. There are ongoing debates over how widespread forest-refugial patches were during the Late Glacial Maximum (13,000-10,000 years ago; Willis and van Andel, 2004; Kaplan et al., 2016). Pollen studies illustrate that parts of southern Europe, notably in the northern Balkans, north of the Alps, and in southern Iberia, tree refugia persisted (Willis et al., 2000). Palynological data also demonstrate that most of central and northern Europe was tree-less and composed of open grasslands (Davis, 1976; Hewitt, 1996, 1999; Tarasov et al., 2000; Petit et al., 2003). Rosaceae, as insect pollinated plants, are less likely to appear in palynological records, and it is unclear how prominent the clade was on these grasslands. Humans (and possibly bear) appear to have taken on the role of primary seed dispersers for the European wild apple since the Neolithic. Although, humans have largely neglected their seed-dispersal duties for the European wild apple during the past 1500 years, since the domesticated apple was introduced to Europe (with the exception of some recent use of hybrids as ornamentals). More recently, humans have contributed to population fragmentation and the loss of other seed dispersers and pollinators.

While populations of the European wild apple are becoming increasingly more fragmentary, far lower rates of gene flow exist in its cousin populations in eastern Kazakhstan (*M. sieversii*) and around the Black Sea (*M. orientalis*). The wild-apple forests of the Tian Shan are highly fragmented and each population is densely packed, the lack of a seed disperser has led to massive range reduction and over competition of sibling lines. Tian Shan wild apples mostly reproduce through shoots and non-sexual propagation today, with essentially no long-distance seed dispersal. The situation for the progenitor of our peach is even worse, most likely the true wild progenitor is extinct, likely due to an inability to support a genetically stable population with large seeds and no proper dispersal agent. Su et al. (2015) note that a true progenitor population has never been identified for peaches, but they present finds of large fossilized peach pits. The fossil endocarps come from a late Pliocene sedimentary formation in Kunming, Yunnan, southwestern China and date to roughly 2.6 M years ago. The authors refer to them as *P. kunmingensis* and the fossils clearly illustrate the fact that peaches evolved to be too large for bird or small-mammal dispersal before the Pliocene. Other large-seeded *Prunus* spp. exist across Eurasia with a diversity in the southern Himalaya, including *P. davidiana, P. mira, P. mongolica*, and cultivated and feral *P. persica*. Some examples of large-seeded *P. mira* have survived with limited seed dispersal through natural cloning, shoot propagation, and extensive life expectancies, examples of millennium-old trees grow in Linzhi County, eastern Tibet (Wang and Zhuang, 2001).

## Domestication

### Pre-domestication Interactions

Many scholars have suggested that genetic bottlenecks accompany human-driven selection during early cultivation, with subsequent diversification into landraces (Doebley et al., 2006; Slotte et al., 2013). However, these domestication processes do not play out in tree crops (McKey et al., 2010; Miller and Gross, 2011; Myles et al., 2011; Gross et al., 2012, 2013; Gaut et al., 2015; Velasco et al., 2016). While some studies show low genetic diversity in cultivated peaches, this may reflect the general loss of the true progenitor population. In addition, the loss of diversity likely predates domestication, as large-seeded wild peaches lost range and their populations reduced following the Pleistocene (Velasco et al., 2016). Almonds (*Prunus dulcis*), which are not dispersed via animal consumption, show a seven-fold higher genome-wide nucleotide diversity when compared to the peach. Some of the diversity in the *Prunus* clade may be explained by a close examination of the mating systems (Velasco et al., 2016). Velasco et al. (2016) suggest that there is a gradual decrease in peach diversity over the past two million years and that wild population sizes have remained very low for the past five millennia.

Some scholars have attempted to place a protracted model of domestication onto the apple (Duan et al., 2017) and to fruit trees more broadly (Fuller, 2017). In some cases, these proposed models for domestication suggest an origin of cultivation dating back 10,000–4,000 years (Duan et al., 2017), despite a complete lack of evidence for farming economies in the Tian Shan until roughly 4,500 years ago and no evidence for the cultivation or maintenance of woody perennials until roughly 2,400 years ago (Spengler et al., 2017). While it is likely that people were actively conserving wild Tian Shan apple trees and possibly even replanting or intentionally spreading the population during the past five millennia, it is unlikely that people were intentionally breeding or directing reproduction in the sense of arboriculture prior to about 3,000 years ago. Likewise, in Europe, where clear evidence of foraging of wild apples dates back to the Mesolithic, there is no solid evidence to support arguments that people were cultivating or breeding wild *M. sylvestris* trees. Even Zvelebil (1994), who argued that humans had a strong selective force on European vegetation communities before the introduction of farming, does not believe that arboriculture was an early cultural adaptation.

**FIGURE 4:**
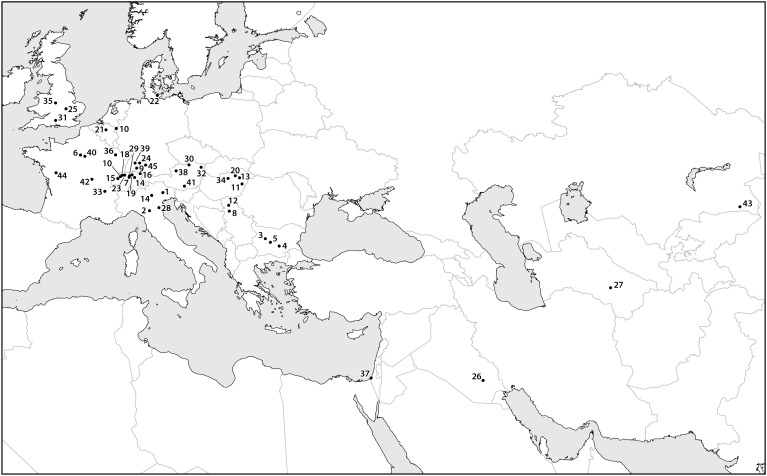
A map containing data points for select finds of archaeobotanical remains of apple seeds recovered from archeological sites dating between the eighth and first millennia B.C. Sites dating to later time periods are assumed to contain remains of cultivated apples, which are likely to have been dispersed beyond their early Holocene distribution. Most of the data points presented in the map cluster over known Pleistocene glacial refugia, illustrating the limited range and areas of density in wild *Malus* during the early and mid-Holocene. The site names, ages, and references are presented in Supplementary Material.

Many scholars have claimed that early Europeans used fire to clear forests and, starting in the Neolithic, left old fields fallow. These practices, in addition to consuming and spreading apple fruits, could have greatly promoted the spread and success of wild *Malus* spp. trees during the Holocene. Several scholars have argued that humans were modifying vegetation using fire during the late Pleistocene (Kaplan et al., 2016), the resulting forest clearings could also have facilitated the spread of wild apples and their relatives. At some archeological sites as far back as the Neolithic in core areas of wild apple populations (i.e., glacial refugial zones), high concentrations of wild apple seeds suggest that humans maintained a very close relationship with these trees, possibly verging on what many people would call cultivation. Some of the best examples come from Neolithic villages in central Switzerland, in these cases tens of thousands of preserved apple seeds were recovered (Antolín et al., 2017). Likewise, the foraging of wild apples in some areas can be traced to the Mesolithic; *Malus* sp. seeds were reported from the site of Staosnaig on Colonsay, Scotland (Carruthers, 2000; Mithen et al., 2001). Additionally, thousands of archaeobotanical finds of seeds that appear to be from European wild apples have been recovered from Neolithic and Bronze Age archeological sites in Europe (Figure 4). Interestingly, these finds seem to concentrate along a strip through the central lateral zone of Europe. This belt reflects the extent of the Pleistocene permafrost and glacial refugial zones of Europe. As I suggested above, before humans began to actively disperse trees in the late Holocene, their range expansion was limited or possibly stationary. The distribution of early archaeobotanical finds seems to suggest that apples did not successfully colonize the opening permafrost steppes of Europe as the Pleistocene ended (Figure 2, 4). There may have been a delay of many millennia before wild European apples colonized Northern Europe. Once people started to collect, spread, and possibly cultivate the trees, they rapidly colonized the rest of Europe.

Humans have been manipulating wild *Malus* spp. populations across Europe and West Asia since long before domesticated hybrids emerged. The heavily cited finds of a string of desiccated halved apples in a royal tomb at Ur, dating to the late fourth millennium B.C., are most likely *M. orientalis* (Ellison et al., 1978; Postgate, 1987; Renfrew, 1987), as are the early first millennium B.C. specimens form Kadesh Barnea in the Levant (Zohary et al., 2012). Stringing and drying the small apples may have helped preserve them, but likely also helped cut the highly tart flavor, by rehydrating the apples through boiling. While modern humans tend not to favor the tart acidic flavor of wild crabapples, other mammals, including Pleistocene megafaunal browsers, are/were attracted to them. Biblical and later Classical references to large sweet apples are the first clear indications of large-fruiting hybrids (see Janick, 2005).

The fruit of *Malus* trees were favored by humans across Asia as well as Europe for millennia before any of them were cultivated. In addition to the importance of *M. orientalis*, a species that contributed genetic material to the modern apple, *M. asiatica* (now lumped into *M. siversii*) and *M. prunifolia* (Willd.) Borkh. have been cultivated in China for at least two millennia (Duan et al., 2017). *M. baccata* (L.) Borkh. is a small-fruiting species, with a wide distribution, and it was likely collected by early people across most of this range. Archaeobotanical seeds identified as *M. baccata* have been reported as far east as the Kali Gandaki Valley, Nepal, where they were recovered from high elevation burials (Knörzer, 2000). *M. baccata* is a small-fruited avian-dispersed species, and, generally speaking, avian-dispersed fruit trees and shrubs have prospered during the Holocene, hence the expansive geographic range of this tree in relation to its large-fruited relatives (Figure 2). Thus far, the only example of a possible archaeobotanical find of an apple seed recovered from the accepted center of origin for the modern apple – the Tian Shan Mountains of Kazakhstan – dates to the end of the first millennium B.C., at the village site of Tuzusai (Spengler et al., 2017).

### Domestication Through Hybridization

The idea that much of the variation we see in fruits under cultivation is a result of hybridization is not new, nor is the idea of rapid domestication through unconscious hybridization resulting from seed dispersal by humans (Sturtevant, 1885). Ugent (1970) hypothesized that much of the variation in domesticated potatoes is largely a result of hybridization and anthropogenically directed gene flow. Likewise, Clarke and Merlin (2013) presented a model for the domestication of psychotropic cannabis based on hybridization of disparate populations across Central Eurasia (see also Small, 2015). Hughes et al. (2007) discuss domestication through hybridization in *Leucaena* Benth., *Agave* L., and *Opuntia* Mill. Hexaploid bread wheat, octaploid strawberries, and the ploidy complex of raspberries (*Rubus* spp.) are all examples of domestication through genome duplication. Other polyploid crops likely followed a similar trajectory, such as bananas (*Musa* L.), citrus (*Citrus* L.), kiwi (*Actinidia* Lindl.), oca (*Oxalis tuberosa* Molina), and peanuts (*Arachis hypogaea* L.) (Hughes et al., 2007). Many genetic clades within Rosaceae represent hybrid complexes, and unintentional human-caused hybridization has led to extensive morphological change leading to many showy ornamentals and fruits, such as the loganberry (*Rubus* × *loganobaccus* L.H. Bailey), boysenberry (*R. ursinus* × *R. idaeus*), or marionberry (*Rubus* sp.). In some cases, such as for *Crataegus*, apomictic complexes even further complicate taxonomy.

Multiple genome-wide genetic studies utilizing large-scale sampling of modern apple populations have demonstrated that our modern apple is a hybrid of several wild species (Harris et al., 2002; Velasco et al., 2010; Cornille et al., 2012). Cornille et al. (2014) laid out in detail how they envision this hybridization process unfolding for apples. Gharghani et al. (2009, 2010) specifically emphasized the important genetic contributions that came from hybridizing *M. sieversii* and *M. orientalis*, the latter of which may have been cultivated or maintained in Iran long before the Tian Shan apple was introduced. However, it is clear that the European wild apple (*M. sylvestris*) also played a significant role in subsequent hybridizations (Cornille et al., 2012). The European wild apples were undoubtedly maintained and possibly cultivated long before *M. sieversii* × *M. orientalis* hybrids made their way to Europe. Therefore, the driving force of apple domestication appears to have been the *trans*-Eurasian crop exchange, or the movement of plants along the Silk Road (Spengler, 2015, 2019).

**FIGURE 5:**
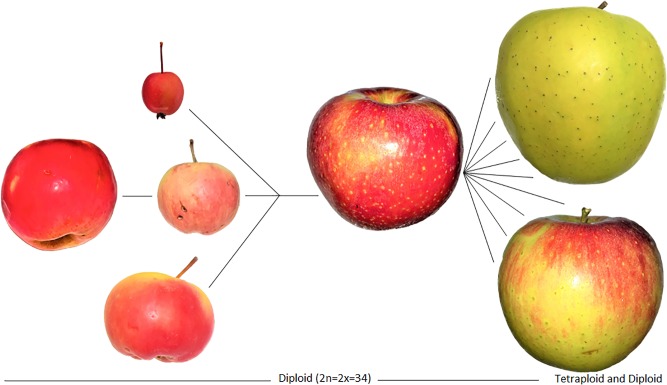
Images of the domestication process for apples, relying on hybridization, and resulting in a domestication complex of *Malus* spp. Genetic data illustrate the introgression of genes from four species; the resulting hybrids were cloned and later diversified. The Tien Shan wild apple (*M. sieversii*) is on the far left, followed by *M. baccata* (top), *M. orientalis* (middle), and *M. sylvestris* (bottom). They collectively hybridized into the modern domesticated apple (*M. pumila/domestica*) on the left which was diversified through cultivation and further hybridization over the past two millennia into thousands of landraces. These hybrids are fixed through grafting and cloning, and today our modern apples reflect at least two millennia of clonal propagation by humans. Note that the specimen of *M. baccata* depicted here is an ornamental and likely hybrid; although, studies show that most wild apples today are the result of considerable crop-to-wild gene flow. Apples on the left of the figures represent the diversification of the domesticated lineage into landraces, likely mostly occurring over the past millennium.

As Cornille et al. (2014:59) state, “[p]eople then took the domesticated apples westwards along the great trade routes known as the Silk Route, where they came into contact with other wild apples, such as *Malus baccata* (L.) Borkh. in Siberia, *Malus orientalis* Uglitz. in the Caucasus, and *Malus sylvestris* Mill. in Europe.” What we think of as the modern apple today is actually a hybrid of at least four separate species. Three of these species maintain large fruits in their progenitor forms, with the Tian Shan apple being the largest of them (Dzhangaliev, 2003). The fourth species has small fruits and is readily dispersed by birds; hence, *M. baccata* maintains a very large range in the wild. The three large-fruiting species have small ranges, relative to their smaller fruited relatives. The range of *M. sieversii* is particularly small and the population is facing extinction. The distinct populations of large-fruiting *Malus* spp. apparently remained isolated since before the Pleistocene. Continental ice served as a physical barrier to gene flow, which was maintained for several millennia after the ice melted. This barrier was only broken when humans began to disperse the fruits over long distances across Europe and Asia. People brought the trees that were already cultivated in the Tian Shan Mountains (presumably no earlier than the first millennium B.C.) into contact with *M. orientalis*. The trees, presumably, were hybridized by insect pollinators, creating robust offspring with traits that people recognized and favored. *M. orientalis* was likely already cultivated or maintained in some areas of far West Asia. It is also likely that now-extinct remnant populations of either of these species existed into the late Holocene, possibly contributing to the domestication process. The hybrids were further dispersed by people, who brought them into the pollination range of the European wild apple. The European wild apple was undoubtedly cultivated by the first millennium B.C. As a result, of the hundreds of thousands of years of genetic isolation, the resulting hybrids expressed greater fitness and likely traits that appealed to humans – possibly larger fruits. This process is illustrated in Figure 5.

### Fixing Hybrid Traits

The traits of this hybrid population were/are not fixed into modern domesticated apples, rather they were obtained and are maintained through cloning. Modern “domesticated” apples are not domesticated in the same way that our annual crops are (see discussion in Cornille et al., 2014). The modern apple is largely a hybrid of wild apples and various landraces. If the seeds of these apples were germinated, the resulting population would express a variety of traits, many of which overlap with the wild progenitor populations (Juniper and Mabberley, 2006; Meyer et al., 2012). Without clonal reproduction by humans, the traits of these hybrids could not have been maintained. Grafting techniques were discussed in the Hippocratic treatise (424 B.C.; Mudge et al., 2009), as well as by Theophrastus (371–287 B.C.), Cato (234–149 B.C.), and Pliny the Elder (23–79 A.D.; Juniper and Mabberley, 2006). Some historians have argued that an account of transporting budwood for grapes, recorded on a cuneiform table, may be a reference to grafting at roughly 3800 years ago (Juniper and Mabberley, 2006). The grafting of apples seems to have been a widespread practice in southern Europe by the early first millennium A.D. (Mudge et al., 2009). Clonally reproduced trees are characterized by lower levels of mutation and selection than their wild counterparts (McKey et al., 2010; Miller and Gross, 2011; Meyer et al., 2012), and most fruit trees experience a mix of cloning and sexual reproduction across their cultivated populations.

Wild Tian Shan apples express a wide range of phenotypic traits and a high degree of plasticity. The wild population has trees that can produce fruits up to 8 cm in diameter and some that have sweet and flavorful fruits. People could simply have selected the best flavored specimens in some ways bypassing the domestication process as it occurred in cereals (Juniper and Mabberley, 2006). Selection for bigger, sweeter, and firmer apples must have occurred rapidly, if we accept that these hybrids formed in the first millennium B.C. The trees that produced preferable fruits were maintained through cloning with minor genetic modification over the past few millennia. Duan et al. (2017) identified alleles associated with fruit sugar content, firmness, color, hormone and secondary metabolic responses, and fruit acidity. Some of the key traits of domestication in the apple include increased yields and reduced masting habits; although, studies have suggested that these traits may be plastic and effected simply by maintaining favorable growing conditions (Dzhangaliev, 2003; Forsline et al., 2003; Goldschmidt, 2013). Apples and peaches are perfect case studies for understanding the role of phenotypic plasticity and hybridization in the domestication process.

These hybrid plants possessed desirable traits, and humans maintained them for thousands of years. Further diversification and the development of landraces over the past millennium or two were driven by limited sexual reproduction. This gene flow is not restricted to apples under cultivation and the process of humans causing gene flow by dispersing apple trees is ongoing across Europe and Asia. These studies demonstrate that traits of domesticated apples are present in essentially all wild apple populations, due to crop-to-wild gene flow.

### Conservation and Continuing Evolution

Several recent studies have noted that most of the trees that ecologists consider “wild” apples today are actually hybrids. Both in Europe and in the Tian Shan, the true progenitor populations show clear genetic signatures of hybridization events. Cornille et al. (2014:380) noted a “large-scale recent introgression from the cultivated apple to *M. sylvestris.*” Crop-to-wild gene flow in apples has been recorded in the Dourdan Forest of France (Cornille et al., 2013, 2015; Feurtey et al., 2017) and across the Rhine Valley at six locations in Germany (Schnitzler et al., 2014). These studies identify wild-growing apples in the European forests that genetically express traits more like domesticated apples. Specific traits of these wild-growing hybrids included larger fruits. The greater the level of introgression of domesticated genes, the larger the fruit (Schnitzler et al., 2014). Over the past few decades, the rate of human-induced seed dispersal has increased due to the sale of ornamental crabapples and commercial apple hybrids. Genetic studies of the commercial crabapples that were sold under the moniker of wild, illustrates that they contained “substantial levels of *M. domestica* ancestry” (Cornille et al., 2013:183). Cornille et al. (2013) took the study a step further and noted that the hybrids express greater fitness in the wild, illustrating the mechanisms that drove larger fruit evolution at each stage of development. The hybrid apples germinated earlier and seedlings grew faster; similar conclusions were drawn a decade earlier by looking at apple hybrids (Larsen et al., 2006).

Pockets of genetic diversity and late Holocene apple breeding zones exist across Europe and South Asia, for example in the Karaman region of Turkey separate landraces are still recognized between many villages. Genetic studies demonstrate that these village apple populations remained isolated from each other and from the broader globalized world (Sönmezoğlu and Kütük, 2014). There is also a genetically recognized diversity among Iranian landraces today; Farrokhi et al. (2011) suggest that this diversity is due to the geographic proximity of the Iranian Plateau to Central Asia. If their assessment is correct, then the greater diversity could be due to continual crossing of wild populations or inflow of new varieties from Central Asia. As discussed earlier in this paper, genetic hot spots in *Malus* occur on glacial refugial zones, suggesting that future germplasm collection campaigns should target these areas. This is important to note seeing that germplasm facilities are not barriers to gene flow and studies of genetic diversity among apple accessions in gene banks demonstrate high rates of first-generation hybrids. A study of the Pometum gene bank collection in Denmark not only identified a high number of first-generation relatives, but also a significant number of clones (Larsen et al., 2018).

## Conclusion

The key for understanding how domestication occurred in rosaceous trees rests in figuring out the evolutionary driver for large fruits in the wild – seed dispersal through megafaunal mammals – and the process of evolution for these large fruits – hybridization (in some cases, resulting in whole genome duplication). The study of domestication in the Rosaceae clade provides an important critique of plant domestication studies broadly, illustrating that there is not a one-size-fits-all model for plant domestication. Notably, protracted models that have been developed for cereal domestication do not hold up for all crops. Domestication through hybridization can be a rapid unconscious human-driven process. Domestication studies often ignore the evolutionary processes leading up to human cultivation. This review illustrates how informative the paleontological record can be in providing parallel examples of evolution. Large fruits in the Rosaceae family appear to have evolved due to hybridization events in the wild and were selected for through the success in recruiting large megafaunal mammals as seed dispersers. While larger fruits are energetically more costly than their smaller counterparts, they were selected for on the late Miocene landscape of Eurasia, allowing trees to respond faster to climate change and environmental stressors.

**FIGURE 6:**
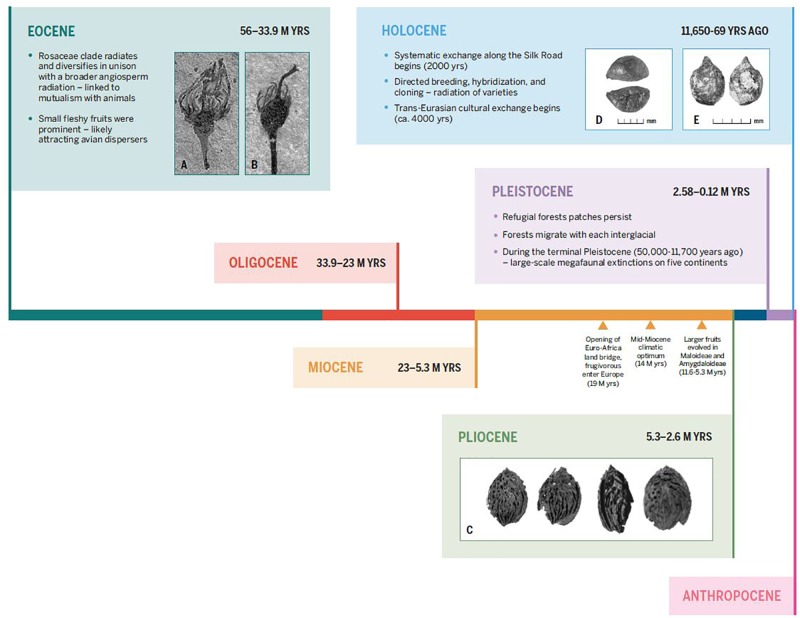
Time scale showing major evolutionary changes in the two main lineages of large-fruiting rosaceous trees, spanning from the Eocene to the present. Key events discussed in the text are indicated. Additionally, inset images depict fossil and subfossil remains of fleshy fruits in Rosaceae; the first set show small fleshy fruits from the Eocene and large fleshy fruits in the Pliocene (which likely evolved by the late Miocene). Inset **(A)** and **(B)**: fossil *Prunus*-like floral/fruit structures, showing inferior ovoid, from the Eocene (ca. 40 M yrs) of North America, from DeVore and Pigg (2007). Inset **(C)**: Pliocene peach endocarps from near Kunming, China, from Su et al. (2015). The second set of images show: **(D)** a large domesticated seed from the Pamir Mountains of Uzbekistan dating to roughly 1000 years ago (Spengler, 2019) and **(E)** an archaeobotanical rosaceous seed from the Tuzusai site in the Tien Shan Mountains of Kazakhstan, dating to roughly 400 B.C. (Spengler et al., 2017).

During the late Miocene and Pliocene, the dispersal of ancestral Rosaceae seeds by frugivorous megafauna allowed disparate populations to hybridize that would have otherwise been outside the reach of pollinators (Figure 6). This hybridization event, or series of events, drove the evolution of larger fruits and diversified the clade. During the late Holocene, the dispersal of *Malus* seeds or saplings across Eurasia by humans, similarly, broke the barriers of genetic isolation and drove further diversification in the clade. Humans were likely interacting with and maintaining wild apples in refugial forests across Europe and West Asia throughout the latter half of the Holocene. It was the formation of the *trans*-Eurasian exchange routes, the Silk Road, that ultimately led to the dispersal of disparate apple populations across two continents (Spengler, 2019) (Figure 6). These ancestral populations had been genetically isolated since before the Pleistocene and appear to have readily hybridized once brought into contact. Humans maintained and propagated these large-fruiting hybrids through cloning and grafting – creating our modern apple. While the apple case study may not be representative for all arboreal crops, it appears to parallel other members of the Rosaceae clade. This study suggests that we cannot use models of crop domestication that have been produced solely on the basis of large-seeded annual grasses as blanket analogies for all crops.

## Author Contributions

RS contributed to the conception and design of the work, as well as drafting the manuscript.

## Conflict of Interest Statement

The author declares that the research was conducted in the absence of any commercial or financial relationships that could be construed as a potential conflict of interest.
