# Supplementary material for: Origins of the Apple: The Role of Megafaunal Mutualism in the Domestication of Malus and Rosaceous Trees
Source: Front Plant Sci. 2019 May 27;10:617. doi: 10.3389/fpls.2019.00617 (PMC6545323; doi:10.3389/fpls.2019.00617)
Supplement: Supplementary file 1 [file Table_1.DOCX]

Supplementary Material

| Country | Site | Classification | Age | New |
| --- | --- | --- | --- | --- |
| Sixth Millennium B.C. | | | |  |
| Italy | Sammardenchia-Cûeis | Malus sp. | Sixth Millennium B.C. | 1 |
| Italy | Bazzarola | Malus sp. | Sixth Millennium B.C. | 2 |
| Bulgaria | Azmak | Malus/Pyrus | Sixth Millennium B.C. | 3 |
| Bulgaria | Dimitrievo | Malus/Pyrus | Sixth Millennium B.C. | 4 |
| Bulgaria | Čavdar | Malus/Pyrus | Sixth Millennium B.C. | 5 |
| Fifth Millennium B.C. | | | |  |
| France | Bercy | Malus sylvestris | Mid-Fifth Millennium B.C. | 6 |
| Switzerland | Cham | Malus sp. | Late Fifth Millennium B.C. | 7 |
| Serbia | Tell Gomolava | Malus sylvestris | Early Fifth Millennium B.C. | 8 |
| Fourth Millennium B.C. | | | |  |
| Germany | Hornstaad-Hörnle | Malus sylvestris | Early Fourth Millennium B.C. | 9 |
| Netherlands | Elsloo | Malus sylvestris | Early Fourth Millennium B.C. | 10 |
| Hungary | Berettyóújfalu-Szilhalom | Malus sylvestris | Fourth Millennium B.C. | 11 |
| Serbia | Gomolava | Malus sylvestris | Fourth Millennium B.C. | 12 |
| Hungary | Tiszapolgár-Csöszhalom | Malus sylvestris | Fourth Millennium B.C. | 13 |
| Italy | Rocca di Manerba | Malus sp. | Fourth Millennium B.C. | 14 |
| Switzerland | Concise sous-Colachoz | Malus sylvestris | Fourth Millennium B.C. | 15 |
| Switzerland | Arbon Bleiche 3 | Malus sylvestris | Mid-Fourth Millennium B.C. | 16 |
| Switzerland | Sutz-Lattringen | Malus sylvestris | Mid-Fourth Millennium B.C. | 17 |
| Switzerland | Nidau | Malus sylvestris | Mid-Fourth Millennium B.C. | 18 |
| Third Millennium B.C. | | | |  |
| Switzerland | Horgen | Malus sylvestris | Early Third Millennium B.C. | 19 |
| Hungary | Szihalom-Földvár | Malus sylvestris | Third Millennium B.C. | 20 |
| Belgium | Vaux-et-Borset | Malus sylvestris | Third Millennium B.C. | 21 |
| Germany | Wangels | Malus sp. | Third Millennium B.C. | 22 |
| Switzerland | Saint Aubin | Malus/Pyrus | Third Millennium B.C. | 23 |
| Germany | Bad Buchau | Malus sylvestris | Third Millennium B.C. | 24 |
| United Kingdom | Kingsmead Quarry | Malus sylvestris | Third Millennium B.C. | 25 |
| Iraq | Ur | Malus sp. | Late Third Millennium B.C. | 26 |
| Second Millennium B.C. | | | |  |
| Turkmenistan | Gonur | Cf. Malus | Early Second Millennium B.C. | 27 |
| Italy | Canàr | Malus sylvestris | Second Millennium B.C. | 28 |
| Switzerland | Cham-Oberwil | Malus sylvestris | Second Millennium B.C. | 29 |
| Austria | Thunau am Kamp | Malus sp. | Late Second Millennium B.C. | 30 |
| United Kingdom | Potterne | Malus sylvestris | Late Second Millennium B.C. | 31 |
| Austria | Stillfried | Malus/Pyrus | Late Second Millennium B.C. | 32 |
| France | Lac du Bourget | Malus sylvestris | Late Second Millennium B.C. | 33 |
| Hungary | Luas, Varjú dülö | Malus sylvestris | Late Second Millennium B.C. | 34 |
| First Millennium B.C. | | | |  |
| United Kingdom | Arrow Valley | Malus sylvestris | Early First Millennium B.C. | 35 |
| France | Moselle Valley | Malus sp. | First Millennium B.C. | 36 |
| Israel | Kadesh Barne’a | Malus sp. | First Millennium B.C. | 37 |
| Austria | Hallstatt | Malus sp. | Mid-First Millennium B.C. | 38 |
| Germany | Hohmichele | Malus sylvestris | Mid-First Millennium B.C. | 39 |
| France | Bussy-Saint-Georges | Malus sylvestris | Mid-First Millennium B.C. | 40 |
| Austria | Goldes | Malus sylvestris | Mid-First Millennium B.C. | 41 |
| France | Chalon-sur-Saône | Malus domestica | Late First Millennium B.C. | 42 |
| Kazakhstan | Tuzusai | Malus/Pyrus | Late First Millennium B.C. | 43 |
| France | Mazières-en-Mauges | Malus domestica | Late First Millennium B.C. | 44 |
| Germany | Forggensee | Malus sp. | Late First Millennium B.C. | 45 |

**
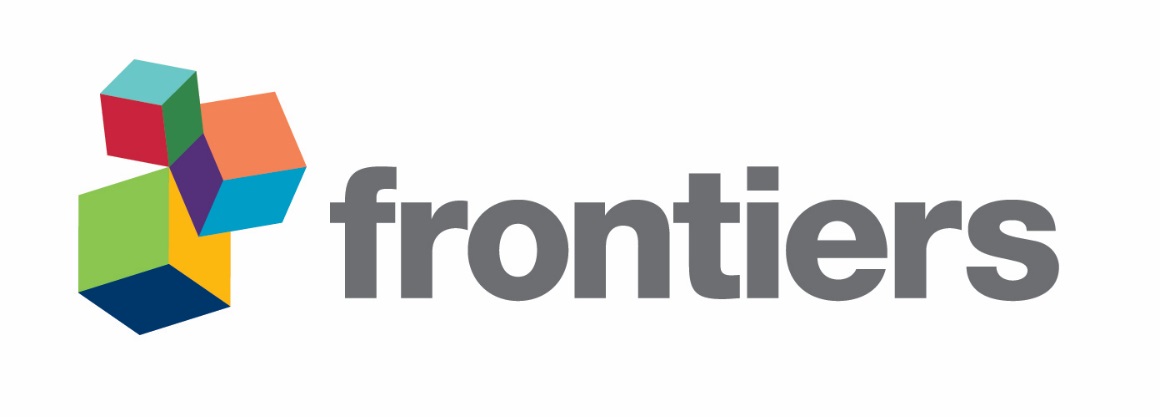
**

**Supplementary Figure 1.** This table correlates to the data points as presented in Figure 4, and represents a selection of sites chronologically ranging between the sixth and first millennia B.C. that provided archaeobotanical remains of apples. These references have been compiled from many sources, notably a database compiled by Helmut Krall.

1. Rottoli, M. (1999) I resti vegetali di Sammardenchia-Cûeis (Udine), insediamente del neolitico antico. In: Ferrari A/Pessina A (eds) Sammardenchia-Cûeis. Contributi per la conoszenza di una comunità del primo neolitico. Comune Udine, Mus Friul Storia Nat pubbl 41. Udine, pp 307-326
2. Carra M. (2000) Agricoltura ed economia di sussistenza del territoria reggiano della Preistoria. Studio paleocarpologico perliminare dell’insediamento neolitico di Bazzarola (RE). In: Macellari R / Tirabassi J (eds) Pagine di archeologogia - studi i materiali. Reggio Emilia, pp 1-74
3. Marinova, E. (2007) Archaeobotanical data from the early Neolithic of Bulgaria. In Sue Colledge and James Conolly (Eds.) The origins and spread of domestic plants in southwest Asia and Europe. (pp.93-109) Left Coast Press: Walnut Creek, California.
4. Marinova, E. (2007) Archaeobotanical data from the early Neolithic of Bulgaria. In Sue Colledge and James Conolly (Eds.) The origins and spread of domestic plants in southwest Asia and Europe. (pp.93-109) Left Coast Press: Walnut Creek, California.
5. Marinova, E. (2007) Archaeobotanical data from the early Neolithic of Bulgaria. In Sue Colledge and James Conolly (Eds.) The origins and spread of domestic plants in southwest Asia and Europe. (pp.93-109) Left Coast Press: Walnut Creek, California.
6. Dietsch-Sellami, M.-F. (2001) Les plantes sauvages au menu des chasséens de Bercy: l’apport de la carpologie In: Actes des Journées archéologiques d’Île-de-France. 9 et 10 décembre 2000 (eds Garcia D / Vallet L) [8 pp]
7. Martinoli, D. and S. Jacomet (2002) Pflanzenfunde aus Cham-Eslen. Erste Ergebnisse zur Versorgung mit pflanzlichen Nahrungsmitteln In: Gross-Klee E / Hochuli S, Die jungsteinzeitliche Doppelaxt von Cham-Eslen Tugium 18, 2002, 69-101 (esp pp 76-77)
8. van Zeist, W. (2003) Plant husbanry and vegetation of Tell Gomolava, Vojvodina, Yugoslavia. Palaeohistoria 43/44, 2001-2002, 87-115
9. Maier, U. (2001) Untersuchungen in der neolithischen Ufersiedlung Hornstaad-Hörnle 1A am Bodensee. In: Maier U / Vogt R, Siedlungsarchäologie im Alpenvorland 6. Botanische und pedologische Untersuchungen zur Ufersiedlung Hornstaad-Hörnle 1A. Forsch Ber Vor- u Frühgesch Baden-Württemberg 74. Stuttgart, pp 9-233 pl 1-50 tab 1-17 [more than 370 pp!]
10. Bakels, C. C. (2001) Verkoolde zaden uit de bandkeramische nederzetting Elsloo-Sanderboutlan. Archeol Limburg 87: 18-23
11. Gyulai, F., 2010. Archaeobotany in Hungary: Seed, Fruit, Food and Beverage Remains in the Carpathian Basin from the Neolithic to the Late Middle Ages. Budapest: Archaeolingua.
12. Van Zeist, W., 2003. Plant husbandry and vegetation of tell Gomolava, Vojvodina, Yugoslavia. Palaeohistoria 43/44: 87-115.
13. Gyulai, F., 2010. Archaeobotany in Hungary: Seed, Fruit, Food and Beverage Remains in the Carpathian Basin from the Neolithic to the Late Middle Ages. Budapest: Archaeolingua.
14. Barfield, H., M. A. Borrello, S. Buteux, and M. Ciararldi (2002) Scavi preistorici sulla Rocca di Manerba, Brescia. In: Ferrari A / Visentini P (eds) Il decline del mondo neolitico. Ricerche in Italia centro-settentrionale fra adpetti peninsulari, occidentali e nord-alpini. Atti del convegno Pordenone 2001. Quad Mus Archeol Friuli Occ 4, 2002, 291-309
15. Märkle, T. (2000) Die Wildpflanzen der Cortaillod-moyen-zeitlichen Besiedlung von Concise-sous-Colachoz, Kt. Waadt, Schweiz. Archäol Inf 23,2: 277-280
16. Hosch, S. and S. Jacomet (2001) New aspects of archaeobotanical research in central European Neolithic lake delling sites. Environm Archaeol 6: 59-71
17. Brombacher, C. (2000) Archäobotanische Untersuchungen. In: Hafner A / Suter PF (eds) –3400. Die Entwicklung der Bauerngesellschaften im 4. Jahrtausend v. Chr. am Bielersee aufgrund der Rettungsgrabungen von Nidau und Sutz-Lattringen. Schrr Erziehungsdir Kanton Bern. Bern, pp 155-168
18. Brombacher, C. (2000) Archäobotanische Untersuchungen. In: Hafner A / Suter PF (eds) –3400. Die Entwicklung der Bauerngesellschaften im 4. Jahrtausend v. Chr. am Bielersee aufgrund der Rettungsgrabungen von Nidau und Sutz-Lattringen. Schrr Erziehungsdir Kanton Bern. Bern, pp 155-168
19. Favre, P. (2002) Archäobotanik. In: Achour-Uster C / Eberli U / Ebersbach R / Favre P, Die Seeufersiedlungen in Horgen. Die neolithischen und bronzezeitlichen Fundstellen Dampfschiffsteg und Scheller. Monogr Kantonsarchäol Zürich 36- Zürich - Egg, pp 150-181; ref end vol pp 245-247
20. Gyulai, F., 2010. Archaeobotany in Hungary: Seed, Fruit, Food and Beverage Remains in the Carpathian Basin from the Neolithic to the Late Middle Ages. Budapest: Archaeolingua.
21. Heim, J. and A. Hauzeur (2002) Paysage paléobotanique des sites du Rubané et du groupe de Blicquy à Vaux-et-Borset “Gibour” (Hesbaye, Belgique). Culture du blé nu et récolte de pommes en contexte blicquien. Soc préhist française 99: 289-305
22. Kroll, H. (2001) Der Mohn, die Trichterbecherkultur und das südwestliche Ostseegebiet. Zu den Pflanzenfunden aus der mittelneolithischen Fundschicht von Wangels, Kr. Ostholstein. In: Kelm R (ed) Zurück zur Steinzeitlandschaft. Archäobiologische und ökologische Forschung zur jungsteinzeitlichen Kulturlandschaft und ihrer Nutzung in Nordwestdeutschland [3rd Albersdorf Coll] Heide, 174 pp, pp 70-76
23. Akeret, Ö and I. Geith-Chauvière (2003) Les macrorestes végétaux. In: Wüthrich S, Sait-Aubin / Derrière la Croix. Un complexe mégalithique durant le Néolithique moyen et final. Archéol neuchâteloise 29. Neuchâtel, pp 281-293; 341-354 [all ref]
24. Herbig, C. (2003) Archäobotanische Untersuchungen In: Bauforschung, Phosphatkartierung und botanische Untersuchungen in den Häusern der Moorsiedlung Torwiesen 2 im Federseeried, Bad Buchau, Kreis Biberach Archäol Ausgr Baden-Württemberg 2002, 51-53
25. Chaffey G, Brook E (2012) Domesticity in the Neolithic: excavations at Kingsmead Quarry, Horton, Berkshire. In: Anderson-Whymark H, Thomas J (eds) Regional perspectives on Neolithic pit deposition: beyond the mundane. (Neolithic Studies Group Seminar Papers 12) Oxbow, Oxford, pp 200–215
26. Ellison, R., J. Renfrew, D. Brothwell and N. Seeley, "Some food offerings from Ur, excavated by Leonard Woodley, and previously unpublished," Journal of Archaeological Science, vol. 5, pp. 167-177, 1978.
27. N. F. Miller, "Agricultural development in western Central Asia in the Chalcolithic and Bronze Ages," Vegetation History and Archaeobotany, vol. 8, pp. 13-19, 1999.
28. Castiglioni, E., S. Motella de Carlo, and R. Nisbet (1998) Indagini sui resti vegetali macroscopici a Canàr. In: Balista C/Bellintani P (eds) Canàr di San Pietro Polesine. Recerche archeo-ambientali sul sito palafitticolo. Padusa Quaderni 2. Rovigo, pp 115-130
29. Zibulski, P. (2001) Archäobotanische Untersuchung der Makroreste (Samen, Früchte, und Dreschreste). In: Gnepf Horisberger U / Hämmerkle S, Cham-Oberwil, Hof (Kanton Zug). Befunde und Funde aus der Glockenbecherkultur und der Bronzezeit. Antiqua 33. Basel, pp 150-166; 285-295 [cat]; 333-339 [pl 36-42]
30. Popovtschak, M. and K. Zwiauer (2004) Thunau am Kamp - eine befestigte Höhensiedlung. Archäobotanische Untersuchungen urnenfelderzeitlicher bis frühmittalterlicher Befunde. Österr Akad Wiss Phil-Hist Kl, Mitt Prähist Komm 52. Wien, 278 pp
31. Carruthers, W. J. (2000) Mineralised plant remains. In: Lawson AJ, Potterne 1982-5: Animal husbandry in later prehistoric Wiltshire. Wessex Archaeol Rep 17. Salisbury, pp 72-84; 302-306
32. Kohler-Schneider, M. (2001) Verkohlte Kultur- und Wildpflanzenreste aus Stillfried an der March als Spiegel spätbronzezeitlicher Landwirtschaft im Weinviertel, Niederösterreich. [Österreich Akad Wiss Phil-Hist Kl] Mitt Prähist Komm 37. Wien, 226 pp
33. Bouby, L. and Y. Billaud (2001) Économie agraire à la fin de l’âge du Bronze sur les bords du lac du Bourget (Savoie, France) Comptes Rendus Acad Sci Paris. Sci terre et planètes 333, 201, 749-756
34. Gyulai, F., 2010. Archaeobotany in Hungary: Seed, Fruit, Food and Beverage Remains in the Carpathian Basin from the Neolithic to the Late Middle Ages. Budapest: Archaeolingua.
35. Moffett, L . and Ciaraldi, M. (2000) Plants and economy methodology pp 18-20; Charred plant remains [prehistoric period] pp 32-34; [late Bronze Age] pp 52-53; [late Iron Age] p 59; [Romano-British occupation] pp 162-171; [Late Iron Age, Anglo-Saxon settlement] pp 207-208 In: Palmer SC, Archaeological excavations in the Arrow Valley, Warwickshire. Trans Birmingham Warwickshire Archaeol Soc 103 (for 1999)
36. de Hingh, A. E. (2001) Food production and food procurement in the Bronze Age and early Iron Age. The organisation of a diversified and intensified agrarian system in the Meuse-Demer-Scheldt region (The Netherlands and Belgium) and the region of the river Moselle (Luxemburg and France). Archaeol stud Leiden Univ 7. Leiden, 235 pp
37. Zohary, D., M. Hopf and E. Weiss, Domestication of plants in the Old World: The origin and spread of domesticated plants in Southwest Asia, Europe, and the Mediterranean basin, fourth ed., Oxford: Oxford University Press, 2012.
38. Boenke, N. (2001) Pflanzliche Funde aus dem keltischen Salzbergbau - Hinweise auf die Ernährungsgrundlage. In: Daim F/Kühtreiber T (eds) Sein & Sinn / Burg & Mensch. Kat Niederösterreichisches Landemus NF 434. St. Pölten, pp 208
39. Goppelsröder, A. and M. Rösch (2002) Pflanzliche Funde aus dem keltischen Grabhügel Hohmichele, Gemeinde Altheim (Kreis Biberach) In: Kurz S / Schiek S, Bestattungsplätze im Umfeld der Heuneburg Forsch Ber Vor- Frühgesch Baden Württemberg 87. Stuttgart, pp 163-203
40. Matterne, V. (2002) Les paléosemences. In: Buchez N / Gransar F / Matterne V / Pernaud J-M / Yvinec J-H, L’habitat de La Tène ancienne sur la Z.A.C. Centre ville de Bussy-Saint-Georges (Seine-et-Marne) 2e partie. Rev Archéol Centre France 41, 2002, 35-55 [esp pp 47-55]
41. Oeggl, K. (2003) Botanik. In: Hack S, Der Wiesenkaisertumulus Nr. 4, eine hallstattzeitliche Bestattung in Goldes, Steiermark. Fundber Österreich 41, 2002, 91-165 [esp p 135]
42. Marinval, P. (2000) Économie végétale à l’âge du Bronze final et à l’époque romaine en bord de Saône. In: Bonnamour L (ed) Archéologie des fleuves et des rivières. Paris, pp 48-52
43. R. N. Spengler III, N. F. Miller, R. Neef, P. A. Tourtellotte and C. Chang (2017) Linking Agriculture and Exchange to Social Developments of the Central Asian Iron Age, Journal of Athropological Archaeology, 48: 295-308.
44. Ruas, M.-P. (2000) Chapitre 2. Cultures, importations et alimentation d’après les semences. In: Berthaud G (ed) Mazières-en-Mauges gallo-romain (Maine-et-Loire). Un quartier à vocation artisanal et domestique. Ort, pp 199-257
45. Küster, H. (1999) Pflanzenreste vom Brandopferplatz im Forggensee, Gemeinde Schwangau. In: Zanier W, Der spätlatène- und römerzeitliche Brandopferplatz im Forggensee (Gde. Schwangau). Münchner Beitr Vor- u Frühgesch 52. München, pp 159-161
